# An exploratory study on equity in funding allocation for essential medicines and health supplies in Uganda’s public sector

**DOI:** 10.1186/s12913-016-1698-6

**Published:** 2016-08-30

**Authors:** Donna Kusemererwa, Anita Alban, Ocwa Thomas Obua, Birna Trap

**Affiliations:** 1 Management Sciences for Health, Plot 15, Princess Anne Drive, Bugolobi, P.O. Box 71419, Kampala, Uganda; 2 Faculty of Health Sciences, Department of International Health, University of Copenhagen, Copenhagen, Denmark; 3 Ministry of Health, Pharmacy Division, Lourdel Road, Wandegeya, Kampala, Uganda

**Keywords:** Horizontal equity, Vertical equity, Equity, Essential medicines, Medicines funding, Resource allocation, Uganda

## Abstract

**Background:**

To ascertain equity in financing for essential medicines and health supplies (EMHS) in Uganda, this paper explores the relationships among government funding allocations for EMHS, patient load, and medicines availability across facilities at different levels of care.

**Methods:**

We collected data on EMHS allocations and availability of selected vital medicines from 43 purposively sampled hospitals and the highest level health centers (HC IV), 44 randomly selected lower-level health facilities (HC II, III), and from over 400 facility health information system records and National Medical Stores records. The data were analyzed to determine allocations per patient within and across levels of care and the effects of allocations on product availability.

**Results:**

EMHS funding allocations per patient varied widely within facilities at the same level, and allocations per patient between levels overlapped considerably. For example, HC IV allocations per patient ranged from US$0.25 to US$2.14 (1:9 ratio of lowest to highest allocation), and over 75 % of HC IV facilities had the same or lower average allocation per patient than HC III facilities. Overall, 43 % of all the facilities had optimal stock levels, 27 % were understocked, and 30 % were overstocked. Using simulations, we reduced the ratio between the highest and lowest allocations per patient within a level of care to less than two and eliminated the overlap in allocation per patient between levels.

**Conclusions:**

Inequity in EMHS allocation is demonstrated by the wide range of funding allocations per patient and the corresponding disparities in medicines availability. We show that using patient load to calculate EMHS allocations has the potential to improve equity significantly. However, more research in this area is urgently needed.

**Trial registration:**

The article does not report any results of human participants. It is implemented in collaboration with the Uganda’s Ministry of Health, Pharmacy Division.

**Electronic supplementary material:**

The online version of this article (doi:10.1186/s12913-016-1698-6) contains supplementary material, which is available to authorized users.

## Background

For a health system to be equitable, essential health care must be provided according to need [1–6]. Equity in health care requires that the government health system not only prevents and treats diseases, but ensures equitable access to health care products and services, that is, vulnerable populations that need more get more [3–5]. The Government of Uganda’s national drug policy underscores three important principles of quality of care, equitable and efficient use of available resources, and adequate availability of affordable EMHS at all times [7].

**Fig. 1:**
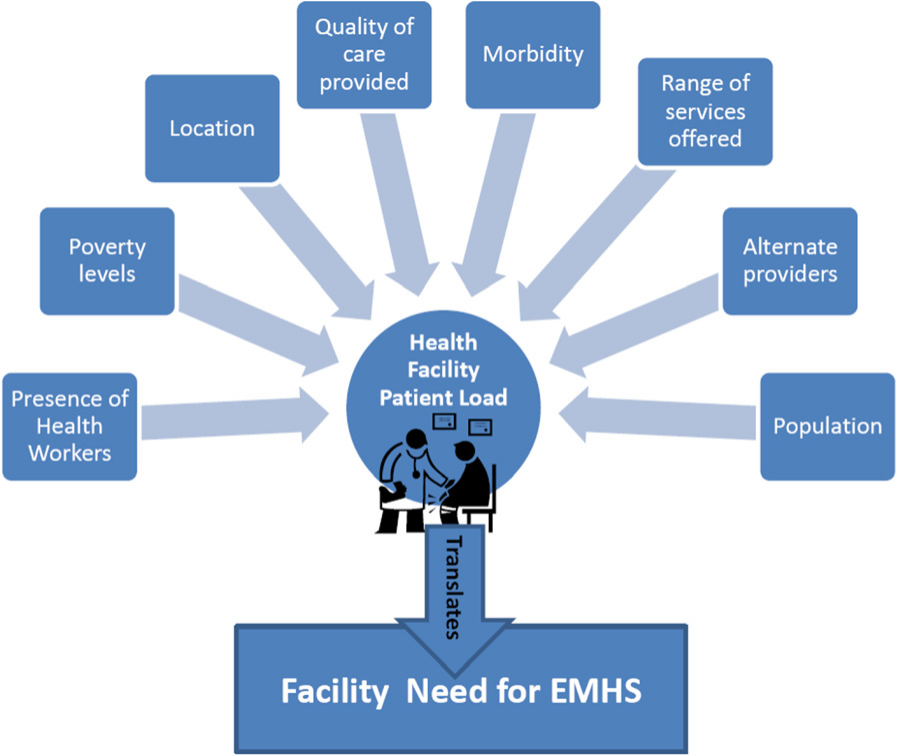
Factors influencing EMHS need in a health facility. Population, morbidity levels, poverty, perceptions about quality of care, availability of medicines and health workers all influence the number of patients visiting a health facility, and as such, the facility’s EMHS needs

A facility’s EMHS needs will be a function of characteristics that include level of care; number and skills of health workers; types, quantity, and quality of services provided; and population characteristics such as size, disease burden, socioeconomic status, and health care-seeking behavior (Fig. 1) [6]. The influence of these factors, and therefore the facilities’ EMHS requirements, differ among facilities at the same level of care and between facilities at different levels of care. These product requirements are a proxy for need, and the facility’s capacity to meet this need will depend to a great extent on its funding allocation.

Equity can be assessed in two ways: horizontally and vertically [8]. We define *horizontal equity* to mean that patients with same needs receive the same treatment and *vertical equity* to mean that patients with greater needs receive more health care than those with lesser needs. Although influenced by the health system’s referral structure and peoples’ care-seeking behavior, generally, patients with more health care needs should be treated at higher-level facilities that can provide increasingly specialized treatments.

Existing information on facility EHMS allocations and patient loads in Uganda points to large differences in the ability of facilities within the same level of care to respond to patient needs and ensure EMHS availability. However, no systematic studies have investigated or documented equity in Uganda’s EMHS funding allocations.

Uganda’s population in 2012/13 was about 35.4 million with a growth rate of 3.2 % per year [9]. Life expectancy in Uganda is 54.5 years; mean number of years of schooling is 4.7, and per capita income is US$497 [10]. Poverty levels are higher in rural rather than in urban areas and the worst in the northern part of the country. Communicable diseases are the leading cause of morbidity, with malaria accounting for 37 % of all outpatient visits in 2012/13 [11]. The primary causes of mortality in children are malaria (28 %) pneumonia (15 %), and anemia (10 %) [12].

Administratively, Uganda is divided into 112 districts, which are further divided into counties, sub-counties, and parishes. In 2013, the country had 5,229 health facilities, of which 55 % were government-owned, 17 % were private not-for-profit, and 28 % private for-profit [13]. The public health system structure includes national referral hospitals, regional referral hospitals, general or district hospitals, health centers (HC) IV, HC III, HC II, and village health teams (HC I). HC I to HC IV services are categorized as primary care. General hospitals and the health centers report to district governments, while the referral hospitals are under central government oversight [5]. The referral system is not well implemented in Uganda, and people commonly seek care at higher level facilities first, where the treatment cost per patient is much greater compared with comparable treatment provided at lower-level facilities.

Most Ugandans seek health care in the public sector resulting in caseloads that are almost five times those of private for-profit providers—10.0 outpatients per provider per day compared with 2.2 for private providers. In addition, the caseloads of public sector rural practitioners are more than twice those in urban areas [14]. One of the key challenges is insufficient human resources, with up to 37 % of health worker posts in the public sector going unfilled [11].

Every year, Uganda’s Parliament allocates funding to procure EMHS through a budget vote. The Ministry of Health negotiates with the Ministry of Finance on the annual budget. The National Medical Stores, the parastatal organization responsible for procurement, storage, and distribution of EMHS to government health facilities, is responsible for carrying out resource allocation decisions. The Government of Uganda funds EMHS through Vote 116, which is an account that the Ministry of Health established at the National Medical Stores to ensure effective and efficient use of funds. Vote 116 is managed so that resources are allocated to a number of vote outputs, including medicines for different purposes. For example, in 2013/14, Vote 116 funds were allocated as follows: 45 % went to medicines and commodities for tuberculosis, malaria, and HIV/AIDS; 16 % for medicines used at specialized institutes as well as reproductive health commodities and emergency medicines; and the remaining 39 % for EMHS used at HC II-IV and general and referral hospitals. In 2013/14, funds for EMHS (excluding HIV/AIDS, malaria, and tuberculosis products) amounted to US$0.99 per capita [15].

This study focuses on the equity of Vote 116 allocations to health facilities for EMHS. The National Medical Stores has the authority to suggest changes to the prescribed allocation of resources at the start of each financial year, but the decision rests with the Ministries of Health and Finance. Public sector facilities receive an annual EMHS allocation as a budget line to draw upon. All facilities at one level, such as HC IVs, receive the same EMHS allocations, and EMHS allocations increase with each level of care. There are three EMHS allocation bands for regional referral hospitals. The vote 116 allocation is the only funding for EMHS available to the health facilities as there are no cost recovery mechanisms in place and the EMHS are provided free of charge to the patients.

EMHS supply to higher-level facilities (HC IVs and hospitals) is organized as a “pull” or order-based system, while lower-level facilities (HC II and III) have a “push” or kit-based system. In the pull system, facilities prepare EMHS orders every other month on the basis of their needs and on the available EMHS allocation in the budget line at the National Medical Stores. HC II and HC III facilities receive pre-packed EMHS kits equivalent to the value of their EMHS allocation every other month—one kit for HC IIs and a larger, more diversified kit for HCIIIs. The HC III kit is more than double that of the HC II kit, both in the quantity of medicines and supplies and in value. In the past, Uganda’s pharmaceutical supply system has varied from entirely order-based to having combinations of pull and push systems. A pull system is more cost-effective, but it requires considerable stock management and quantification capacity within the facilities; whereas, push distribution does not rely on staff ability to quantify, order, or track their expenditures. After EMHS availability became a chronic problem, the government re-introduced the current kit system in HC II and HC III facilities in June 2010 [16].

## Methods

### Aim

This paper examines equity of EMHS funding allocations by exploring the relationships between EMHS allocation, patient load, and EMHS availability.

### Design

The study is cross-sectional descriptive study based on analysis of questionnaires, survey reports and health records from government health facilities, Ministry of Health and National Medical Stores in Uganda.

### Data collection

To explore equity in EMHS allocation at all levels of care, we used three data collection approaches. First, we obtained information on facility EMHS allocation, patient load (number of visits), and medicines availability from order-based facilities (HC IVs and hospitals) through a questionnaire. Second, we gathered information on EMHS allocation and medicines availability from surveys of kit-based facilities (HCIIs and HC IIIs) in 2012/13 [8]. Third, we collected EMHS allocation information from the National Medical Stores for all facilities for financial year 2013/14 and used it to validate facility data and to simulate more equitable funding allocation options. Patient load information was obtained from the national district health information system.

#### Questionnaire for order-based facilities

We purposively sampled 14 (100 %) regional referral hospitals, 11 (23 %) general hospitals, and 16 (10 %) HC IVs. Four inclusion criteria were used to select general hospitals and HC IVs: health facilities had to 1) be located in the 59 (53 %) districts that had a Supervision Performance Assessment and Recognition Strategy (SPARS) in place to build capacity in medicines management; 2) have a SPARS supervisor on site, who could collect data without additional training or travel expenses; 3) be in the public sector and therefore allocated a Vote 116 budget line for EMHS procurement at the National Medical Stores; and 4) have a minimum performance level in medicines management, as per the latest SPARS performance assessment that measures 25 pharmaceutical system indicators [17].

The facilities’ measured performance had to score at least 12.6 (maximum 25) with a stock management score above 2 (maximum 5) to ensure that the findings would not be biased by poor medicines management capacity. Eleven general hospitals and 44 HC IVs met the inclusion criteria. The 44 HC IVs were stratified based on 2012 annual patient numbers. Of the 37 HC IVs with complete patient load datasets, we chose for the sample eight facilities with the highest patient loads and eight with the lowest to assure that we captured facilities with the highest and lowest EMHS allocations per patient. We emailed a pre-tested questionnaire with closed and open-ended questions to SPARS supervisors and regional pharmacists to fill out using facility service statistics for July 2012 to June 2013. Telephone and email follow-up resulted in a 93 % response rate.

#### Kit survey from HCIII and HCII facilities

The Ministry of Health annual kit surveys in 2012 and 2013 assessed how well the EMHS kit supply system was fulfilling the needs related to content and quantities delivered to HC IIs and HC IIIs randomly selected from 12 districts with equal regional representation. We used data on availability and patient load from 22 HC IIs and 22 HC IIIs from the 2012 kit survey [16]. Facility patient attendance data for eight months were extrapolated to annual estimates.

#### EMHS Vote 116 allocation data and patient attendance data

To simulate alternative allocation options to improve equity in health facilities, we used 2012/13 facility-based inpatient plus outpatient attendance statistics from the national district health information system and correlated them with 2013/14 Vote 116 EMHS allocations and 2012/13 EMHS allocations for the kits obtained from the National Medical Stores. These data were used to determine the range of allocations per patient at various levels of care and to investigate what opportunities exist to optimize the allocation per facility and increase equity. Complete sets of allocation and patient data were available for 400 (23 %) HC IIs, 174 (96 %) HC IVs, and 47 (72 %) general hospitals; therefore, we limited the data simulation to these levels of care.

We calculated per patient allocations for HC IVs and hospitals by multiplying the number of inpatient visits by 3.3 and adding it to the number of outpatients, based on estimates that the cost of EMHS for inpatients is about 3.3 times the costs for outpatients [18, 19].

#### Availability of EMHS

We collected information on the availability of a basket of lifesaving, widely used medicines to treat high-morbidity conditions (pneumonia and diarrhea) that take up significant proportions of the EMHS budget. (Table 1). Malaria, HIV/AIDS, and tuberculosis medicines were excluded because they are not funded using the EMHS allocation. For HC IIs, HC IIIs, and HC IVs and general hospitals the basket included six, 10, and 14 medicines, respectively (Table 1).

**Table 1:** Baskets of vital^a^ and lifesaving medicines used to investigate availability at different levels of care

Medicine Description	HC II	HC III	HC IV and hospital
Amoxicillin 250 mg tablet	✓	✓	✓
Carbamazepine 200 mg	N/A	✓	✓
Ceftriaxone 1 g powder for injection	N/A	N/A	✓
Chloramphenicol 1 g injection	N/A	N/A	✓
Ciprofloxacin 500 mg tablet	✓	✓	✓
Cotrimoxazole 480 mg tablet	✓	✓	✓
Dextrose 5 % infusion	N/A	✓	✓
Insulin Mixtard human 100 IU/Ml	N/A	N/A	✓
Metronidazole 500 mg/100 ml infusion	N/A	N/A	✓
Oral rehydration salts	✓	✓	✓
Phenytoin 100 mg tablet	N/A	✓	✓
Sodium chloride 0.9 % infusion	N/A	✓	✓
Tetracycline eye ointment 1 %	✓	✓	✓
Zinc Sulfate 20 mg tablet	✓	✓	✓

^a^Categorized as vital in the EMHS list of Uganda 2012 and approved for use at the respective level of care for which it was investigated and all levels above. N/A: medicine is not allowed for use at the level of care

Availability information included stock on hand on the day of data collection, average monthly consumption, and stock-out days in a 12-month period in 2012/13, obtained from the stock cards and books.

### Analysis

Using MS Excel, we analyzed the data to explore: 1) horizontal equity using EMHS allocation per patient within the same level of care; 2) vertical equity using EMHS allocation per patient by level of care; 3) health facility ability to meet needs within its EMHS allocation; and 4) options for improving equity through alternative allocation methods and principles.

#### Horizontal equity assessment

Population and health system factors such as health behavior, demographics, geography, urbanization, epidemiology, poverty, EMHS allocations, facility staffing and density, among others will determine health facility patient loads, and as such, the facility’s EMHS needs (Fig. 1). In this study, we used patient load as a proxy for EMHS needs and the EMHS allocation per patient within each level of care as a proxy of horizontal equity, recognizing that patients’ needs vary within the same level of care. The EHMS allocation per patient was determined by dividing the annual facility EMHS allocation by the actual annual patient loads (in HC IVs and hospitals) or by extrapolated patient loads (in HC IIs and IIIs).

#### Vertical equity assessment

The cost of treating a patient should increase with each level of care because of the greater breadth and depth of services at higher levels. We limited our assessment of vertical equity to a comparison of the EMHS allocations per patient between levels of care. Assessing the ideal allocation per patient relative to the requirements at each level of care was beyond the scope of this study.

#### Assessment of ability to meet facility needs within the EMHS allocation

A health facility’s ability to meet its patients’ EMHS needs is closely linked to EMHS availability and is a function of its EHMS allocation. To calculate the months of stock on hand, we divided the availability recorded on the day of the study for each basket item by its average monthly consumption. An item was optimally stocked if the facility had two to five months’ worth of stock on hand, understocked if it had less than two months’, and overstocked if it had more than five months’ worth of stock. For each facility, we calculated the percentage of items in each category of 1) optimally stocked, 2) overstocked, and 3) understocked. Then we calculated the average percentage for each of the three categories for each level of care based on the individual category scores for all facilities within the level of care.

The number of stock-out days in a year (actual or extrapolated) was divided by 365 for each product in the basket and aggregated to calculate the facility’s percentage of time out of stock. To assess the ability of a facility to fulfill its EMHS needs, EMHS allocations and per patient allocation were correlated with the average percent availability.

#### Assessment of options to improving equity through alternative allocation

We carried out simulations to determine the EMHS allocations needed for each facility to ensure a ratio of less than two between the highest and lowest allocation per patient in a facility at a specific level of care. We created patient load ranges or bands within each level of care, for example, five bands for HC IVs and general hospitals. Based on the total EMHS allocation available for the level of care, we proposed an average allocation to each facility within that band. Then, based on each facility’s actual patient load, the allocation per patient was calculated. We listed the highest and lowest per patient allocation for each proposed band and compared their ratios to ensure that they were less than two.

### Ethical approval

Not applicable, no approval is required. The study does not involve human participants, human data, human tissue or animals. Data collection was done by SPARS supervisors and regional pharmacist who are Ministry of Health staff and the data they collected did not include any human subject related data. The data related to medicines budgets, procurement expenditures of medicines by the health facilities and patient load data aggregated for each facility. Therefore no ethical approval or waiver from an ethics committee was required.

## Results

Patient loads for 2012/13 varied considerably between facilities and by level of care, although generally they were higher at higher levels of care. Regional referral hospitals’ annual patient loads ranged from 75,735 to 343,266; general hospitals from 60,508 to 154,766; HC IVs from 7,900 to 67,000; HC IIIs from 7,426 and 20,572, and HC IIs between 5,266 and 16,689.

### Horizontal equity

**Fig. 2:**
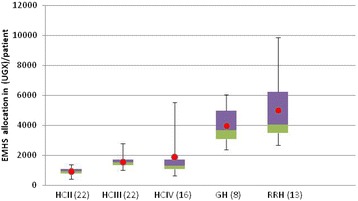
Box-plot depicting EMHS allocation per patient in 2012/13 by level of care*. * Figure showing minimum, maximum, median (*red*), 25th Percentile (*green*) and 75th percentile (*purple*)

Our study documented wide variations in EMHS allocations per patient within the same levels of care. HC IVs and regional referral hospitals had the widest disparities in patient load and therefore in allocation per patient (Fig. 2 and Table 2). At HC IVs, the allocations per patient ranged from 638 to 5,530 Uganda shillings (UGX) (US$0.25–2.14^1^), which is a ratio of 1:9. For regional referral hospitals, the allocations per patient ranged from UGX 2,674 to 9,865 (US$1.04–3.82) with a ratio of 1:4. General hospitals, HC IIIs, and HC IIs had less intra-level variation in allocations per patient with ratios of approximately 1:3: UGX 2,367–6,056 (US$0.92–2.34); UGX1,009–2,795 (US$0.39–1.08); and UGX431–1,367 (US$0.17–0.53), respectively.

**Table 2:** EMHS Allocation per patient^a^ in 2012/13 by level of care

Allocation per patient	HC II (*n* = 22)	HC III (*n* = 22)	HC IV (*n* = 16)	General hospital (*n* = 11)	Regional referral hospital (*n* = 14)
Minimum (UGX)	431	1009	638	2367	2674
Maximum (UGX)	1367	2795	5530	6056	9865
Median (UGX)	922	1521	1328	3700	4041
Mean (UGX)	930	1577	1901	3985	5022
Ratio of means between levels	1.0	1.7	2.0	4.3	5.4
% increase in mean allocation from previous level		70 %	18 %	115 %	26 %
Ratio highest: lowest amount range within level	3.2x	2.8x	8.7x	2.6x	3.7x

^a^ Calculated by annual facility EMHS VOTE 116 allocation divided by annual patient load

### Vertical equity

The annual value of the kit allocation for HC IIs was UGX 7.2 million (US$2,791) and for HC IIIs, it was UGX 20.7 million (US$8,023). HC IVs and general hospitals also got uniform EMHS allocations of UGX 43.2 million (US$16,744) and UGX366.4 million (US$142,015), respectively. The EMHS allocations for regional referral hospitals were more diversified with three levels: UGX 747 million (US$289,535), UGX 900 million (US$348,837), and UGX 1.2 billion (US$465,116), which resulted in a 60 % variation in allocations per patient. The smallest difference in average EMHS allocations per patient was 18 % between HC IIIs and HC IVs. The difference between HC IVs and general hospitals was the largest, with general hospitals having 115 % more in allocations per patient on average than HC IVs (Table 2). We also documented considerable overlap of the allocation per patient between levels (Fig. 2). Most striking was that over 75 % of HC IV facilities had the same or lower allocation per patient than the average per patient allocation for HC IIIs.

### Ability to meet needs within the EHMS allocation

HC IIs reported the lowest (63 %) average availability of vital EMHS on the day of visit and hospitals the highest, with 88 % for general hospitals and 87 % for regional referral hospitals. General hospitals had the shortest average time out-of-stock at 8 %, and HC IVs had the longest average time out-of-stock (28 %) (Table 3).

**Table 3:** Average availability and time out of stock for vital medicines in 2012/13

	HC II (*n* = 22)	HC III (*n* = 22)	HC IV (*n* = 13)	General hospital (*n* = 8)	Regional referral hospital (*n* = 13)
Number of medicines in basket used for measuring availability	6	10	14	14	14
Average % of basket of vital EMHS that were available on day of the visit, independent of the amount available	63 %	77 %	67 %	88 %	87 %
Average % time out of stock for all the items in basket of vital EMHS for the specific level of care, measured as % of stock out days/ year (365 days)	21 %	17 %	28 %	8 %	15 %

**Fig. 3:**
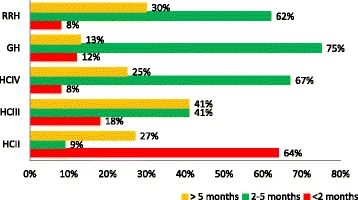
Availability* of vital medicines within each level of care. * Availability measured as percent of facilities that are over (>5 months), under (<2 months) or appropriately stocked (2–5 months)

Overall, 43 % of all the facilities had optimal stock levels, while 27 % were understocked and 30 % were overstocked. Generally, a greater percentage of facilities that placed their own orders had optimal stock levels, while lower-level facilities that received kits were predominantly under- or overstocked (Fig. 3).

**Fig. 4:**
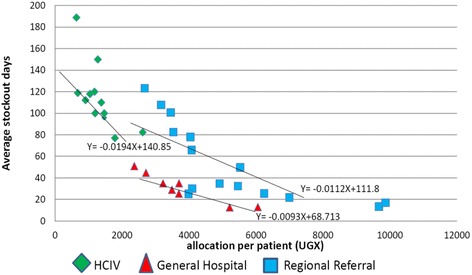
Correlation between stock-out for vital medicines per year and allocation/patient by level of care

Figure 4 shows the robust correlation between annual stock-out days and per patient allocation for the higher-level facilities, which was strongest among HC IV facilities.

### Options for improved equity

#### HC IV and general hospitals

The ratios of the highest and lowest allocations per patient for HC IVs and general hospitals were 1:15 and 1:4, respectively. We simulated allocation per patient based on the overall 2013/14 funding for HC IVs (UGX 9.7 billion, US$3.7 million) and general hospitals (UGX 16.85 billion, US$6.5 million) and estimated patient loads. With the simulation, we could improve horizontal equity by reducing this ratio to less than two for both levels (Tables 4 and 5). The simulation also eliminated the overlap in EMHS allocations per patient between the two levels of care, thereby improving vertical equity.

**Table 4:** Proposed annual allocation bands for HCIV

Annual patient load^a^	Estimated number of facilities	Proposed allocation/ facility million UGX	Allocation/patient		Ratio highest: lowest
			Highest	Lowest	
>60,000	7	130	2,167		
40,000–60,000	10	95	2,375	1,583	
30,000–39,999	56	61	2,033	1,525	
20,000–29,999	94	45	2,250	1,500	
<20,000	7	32		1,600	
Overall	174		2,375	1,525	1.6

^a^(Inpatients*3.3)+ outpatients

**Table 5:** Proposed annual allocation bands for general hospitals

Annual patient load^a^	Estimated number of facilities	Proposed allocation / facility million UGX	Allocation/patient		Ratio highest: lowest
			Highest	Lowest	
>100,000	12	490	4,900		
80,000–99,999	10	400	5,000	4,000	
60,000–79,999	12	350	5,833	4,375	
40,000–59,999	7	235	5,875	3,917	
<40,000	5	150		3,750	
Overall	47		5,875	4,000	1.5

^a^(Inpatients*3.3)+ outpatients

#### HC II

Outpatient visits from over 400 HC IIs that had complete datasets for January 2012 to December 2012 varied from 1012 visits for Bukuumi HC II to 21,565 visits for Kasonga HC II—a 20-fold difference. Given that in 2013/14, each HC II was allocated UGX 7.2 million (US$2,791) to procure EMHS, the allocation per patient range of UGX 334 to 7,115 (US$0.13–2.76), is even wider than the previous year’s results. Based on the simulation, a kit with EMHS to treat priority diseases worth UGX 500,000 (US$194) is suggested, and an HC II would receive between 2 and 31 kits every year depending on its patient load (Table 6).

**Table 6:** Proposed kit allocations for HCII

Annual outpatient attendance	Number of kits^a^	Value / year (UGX)	Allocation/patient (highest for band)
>15,000	31	15,500,000	1033
14000 s	29	14,500,000	1036
13,000 s	27	13,500,000	1038
12,000 s	25	12,500,000	1042
11,000 s	23	11,500,000	1045
10,000 s	21	10,500,000	1000
9,000 s	19	9,500,000	1056
8,000 s	17	8,500,000	1063
7,000 s	15	7,500,000	1071
6,000 s	13	6,500,000	1083
5,000 s	11	5,500,000	1100
4,000 s	9	4,500,000	1125
3,000 s	7	3,500,000	1167
2,000 s	5	2,500,000	1250
1,000 s	3	1,500,000	1500
<1,000 s	2	1,000,000	1000

^a^Annual kit value of 1 is UGX 500.000

## Discussion

Ensuring equity in the allocation of EMHS funding in developing countries is hampered by insufficient funding and poor data quality. As mentioned, many factors influence EMHS need, including disease burden, poverty, and quality of health care, all of which create a complex situation. At one end of the spectrum is the simple “one-size-fits-all” allocation, where all facilities get the same amount, and at the other end, allocation is based on individual facility requirements. It is against this background that we explored equity in EMHS funding allocation and options for improvement. Our sample included both primary care and referral facilities and represented the public health sector’s diversity in terms of patient load, geographical location, and morbidity patterns.

### Data collection

Collecting data was constrained by lack of structured financial data at facility level and data and reporting quality and completeness. To minimize data quality issues, we collected financial and patient data from facilities, from the district health information system, and from National Medical Stores records. Product availability information could only be collected from facilities and depended on how well the facilities were able to manage medicines and place orders. Only higher-level facilities could optimize EMHS availability through placing their own orders because lower-level facilities receive pre-determined kit supplies. By using inclusion criteria linked to SPARS scores, we assured a minimal level of medicines management capacity at the facilities included in the sample. To ensure uniform and high quality data collection, experienced medicines management supervisors collected the data and calculated the average monthly consumption. Patient load and EMHS allocation financial data correlated well with facility information and the national district health information system and the National Medical Stores.

### Allocations per patient

Our study found that EMHS funding allocations to Uganda’s public sector health facilities do not match the facilities’ EMHS needs based on the number of patients that they serve. Patient loads within each level of care vary from 3-fold to as much as 10-fold; however, every facility at the same level receives the same allocation. This means that a sick patient is unlikely to be treated in the same way in two different facilities at the same level of care. Our assessment of vertical equity revealed that referral hospitals’ average EMHS allocation per patient was about five times higher than the average HC II allocation. However, allocations per patient overlap greatly across levels of care and often do not reflect service delivery expectations. For example, the 18 % difference in average allocation per patient for HC IVs compared to HC IIIs is remarkable given the comprehensive services offered at HC IVs and their greater EMHS needs [5]. Although the National Medical Stores took steps to address this anomaly in 2013/14 by increasing the HC IV allocation by 30 %, from UGX 43.2 million to UGX 56 million (US$16,744–21,705), further work needs to determine the best EMHS allocation per patient based on the services to be provided at each level and with the funding available.

### Medicine availability

A facility’s ability to respond to patient needs is directly linked to availability of the medicines required for the specific level of care, which is a function of the facility’s funding allocation and their capacity to place EMHS orders and adequately manage their medicines. Our results found optimal stock levels in hospitals and HC IVs, which likely reflect the fact that facilities that place their own EMHS orders can better identify and meet their actual requirements compared to those facilities that depend on predetermined kits. Although public sector stock-outs can be mitigated by redistributing EMHS, and national redistribution guidelines do exist, it is an expensive option that should be avoided in favor of following needs-based allocation principles.

### A proposed allocation system based on patient load

The Ministry of Health has previously proposed using patient load to determine EMHS funding [20, 21]. We illustrate that with a system based on five different patient load bands for general hospitals, allocations per patient would range from UGX 4,000 to 5,875 (US$1.55–2.28), thus reducing the approximately four-fold variation that currently exists. The HC IV level offers an even bigger opportunity to improve horizontal equity using a five-band system. The resulting allocations there, ranging from UGX 1,525 to UGX 2375 (US$0.59–0.92), would decrease the ratio to less than two compared to the 15-fold variation in the 2013/14 allocations. Restricting the variation between the highest and lowest allocations per patient would also remove the overlap between levels of care and enhance vertical equity. Similarly, the 20-fold variation in patient load at HC IIs has to be addressed by providing kit quantities accordingly, if allocations are to be fair and equitable and variations in stock availability reduced.

In addition to patient load, other factors influence EMHS needs in a facility. Given the present level of system development and the quality of information available, it is difficult to allocate EHMS very precisely. Therefore, a simplified, but not “perfect” allocation system or formula needs to be put in place initially that would improve equity, but allow for some variation. The need for an allocation formula as a means to improve allocation equity has been confirmed in a review study [22].

When limited funding is available for EMHS, as is the case in Uganda with less than US$1 per capita spent annually, the majority of funds must go toward making the most vital supplies available at all facilities. Therefore, the proposed EMHS allocation system minimizes the variations in per patient allocations within the same level of care while maximizing the availability of lifesaving medicines.

Our proposed allocation system has limitations. The EMHS requirements for the same number of patients in a poor, disadvantaged area are bound to be different from those of an urban, affluent area. The risk that the poor are marginalized in a health allocation system always exists, but could be considerable in our recommended system because we have not tried to address the depth of need and disparities in access to care [23, 24]. Other allocation principles have considered additional variables such as infant mortality rate index, hard-to-reach area index, and deprivation index. However, increasing the number of variables also increases the complexity and data needs, and as Fig. 1 illustrates, many of these variables are closely related to patient load. In spite of the limitations, ultimately, using patient load as a proxy for need in situations where data is limited offers a simple and practical solution to a complex issue as well as an incremental improvement over the current system until better data becomes available.

### Additional studies

Studies that address medicines funding allocation and availability in Uganda or similar settings are rare. A systematic search of applicable literature revealed two studies that could shed additional light on the topic [6, 21]. The evidence base for sound decision making on how to increase access to medicines in Uganda, particularly given the financial constraints, is shallow and additional research is needed.

## Conclusions

This study presents evidence of inequity in EMHS allocations to public sector health facilities in Uganda as demonstrated by the wide range of funding allocations per patient at all levels of care and the corresponding disparities in medicines availability. We show that in spite of potential limitations, using patient load as the basis of EMHS allocations in public health facilities has the potential to significantly improve equity in health care service delivery. We also highlight the urgent need for more research in this area.

## Additional file

Additional file 1: The data collection tool is attached in Additional file 1. (DOCX 169 kb)

## Abbreviations

EMHS: Essential medicines and health supplies
HC: Health centers
SPARS: Supervision performance assessment and recognition strategy
UGX: Uganda shillings
USAID: United States Agency for International Development

## Footnotes

1 Based on an exchange rate of 2,584 UGX per US$1.
