# Supplementary material for: An exploratory study on equity in funding allocation for essential medicines and health supplies in Uganda’s public sector
Source: BMC Health Serv Res. 2016 Aug 30;16(1):453. doi: 10.1186/s12913-016-1698-6 (PMC5006492; doi:10.1186/s12913-016-1698-6)
Supplement: Additional file 1: — The data collection tool is attached in Additional file 1. (DOCX 169 kb) [file 12913_2016_1698_MOESM1_ESM.docx]

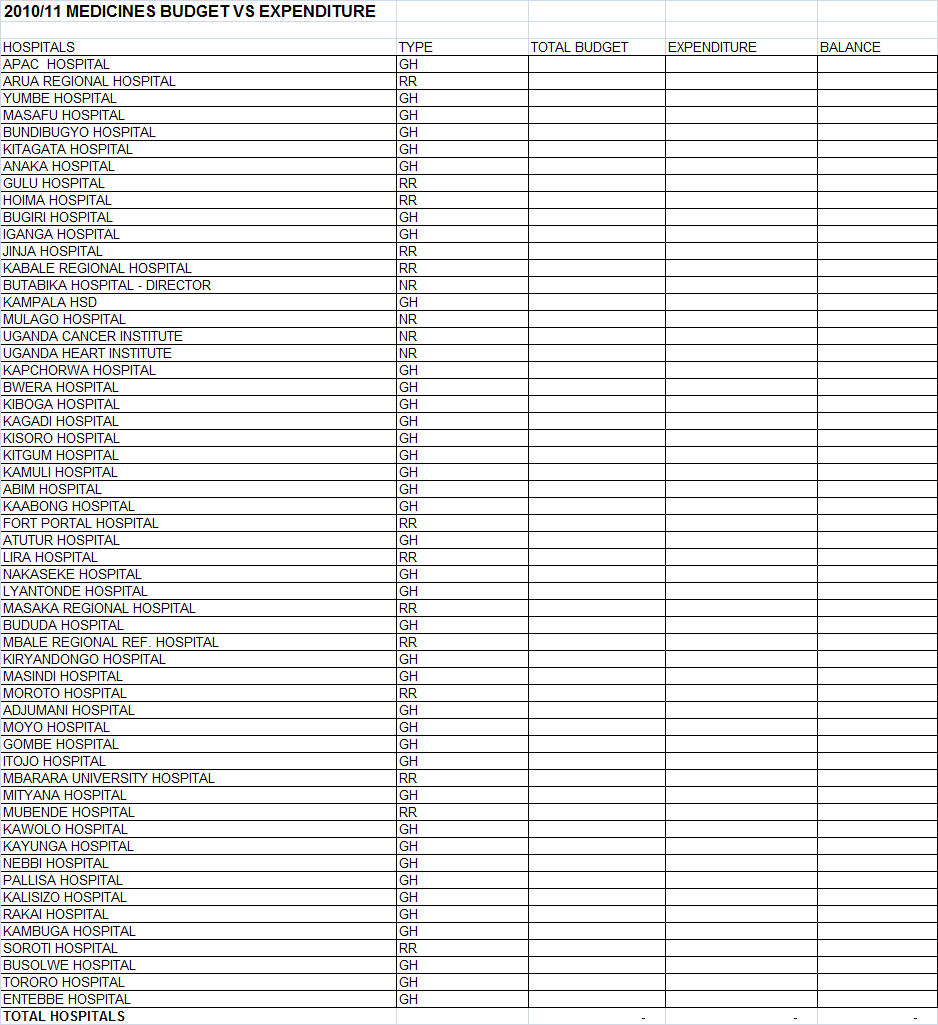


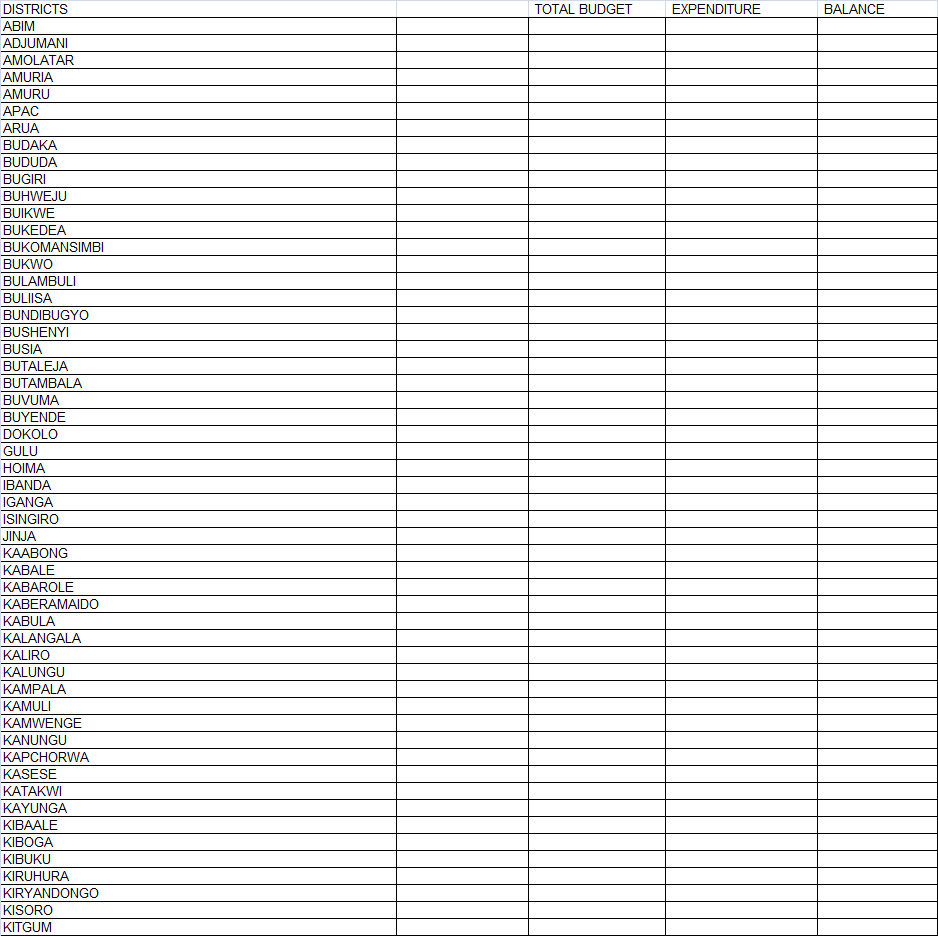


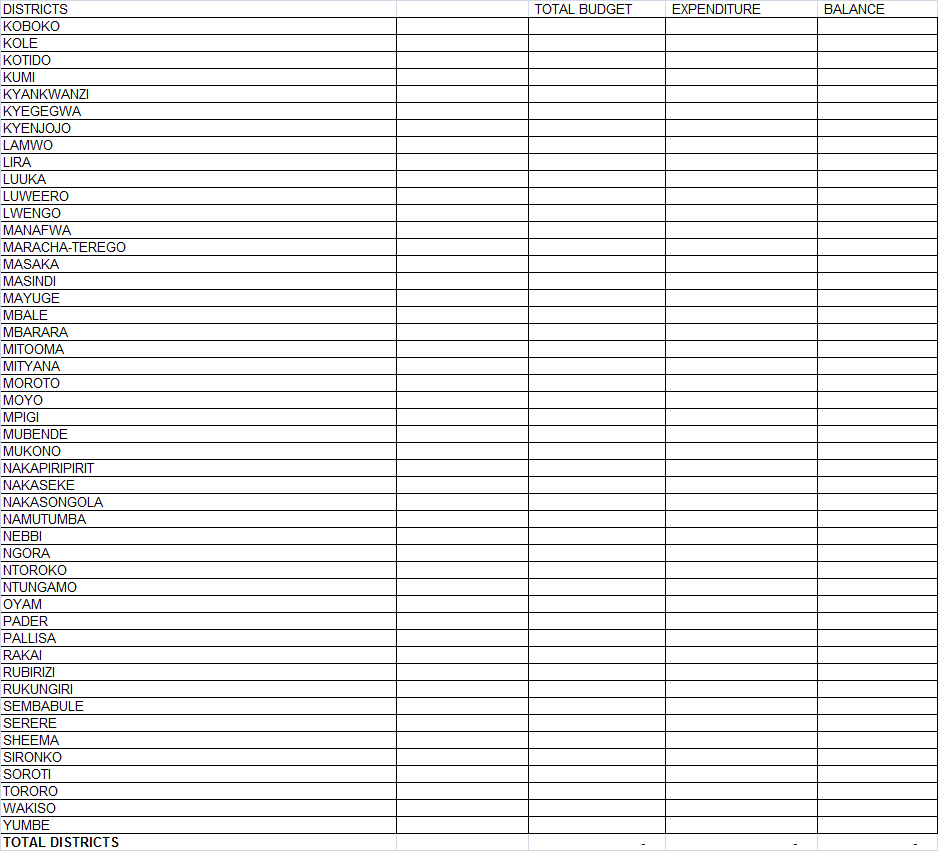


| **Period** | **Organisation unit** | **IPD Admissions** | **IPD Beds Available** | **IPD Patient Days** |
| --- | --- | --- | --- | --- |
|  |  |  |  |  |
|  |  |  |  |  |
|  |  |  |  |  |
|  |  |  |  |  |
|  |  |  |  |  |

| **Period** | **Organisation unit** | **New Attendance - OPD** | **Re-Attendance - OPD** | **Deliveries in unit** |
| --- | --- | --- | --- | --- |
|  |  |  |  |  |
|  |  |  |  |  |
|  |  |  |  |  |
|  |  |  |  |  |
|  |  |  |  |  |
|  |  |  |  |  |
